# Effects of Three Dried *Citrus* Peels as Feed Additives on Growth Performance and Intestinal Microbiota of *Oreochromis niloticus*

**DOI:** 10.1155/anu/5579376

**Published:** 2026-07-09

**Authors:** Yang Yang, Zhanqian Wang, Yifan Hao, Chong Yang, Sisi Xie, Xingchen Li, Nuoying Li, Xiaoxuan Jiang, Wenbin Zhang, Shejian Liang

**Affiliations:** ^1^ College of Life Sciences, South China Agricultural University, Guangzhou, 510642, China, scau.edu.cn; ^2^ School of Pharmaceutical Sciences, Hunan University of Medicine, Huaihua, 418000, China, hnucm.edu.cn

**Keywords:** *Citrus reticulata* “Chachi”, *Citrus reticulata* “Gonggan”, dietary supplementation, growth performance, intestinal microbiota

## Abstract

Citrus processing generates large quantities of peel by‐products that are often discarded, resulting in resource waste and environmental burden. This study systematically evaluated the effects of dietary supplementation with fresh *Citrus reticulata* “Gonggan” peel (NG), fresh *C. reticulata* “Chachi” peel (NC), and 3‐year‐aged *C. reticulata* “Chachi” peel (3 C) on growth performance, muscle quality, antioxidant capacity, immune response, and intestinal microbiota of *Oreochromis niloticus*. Fresh peels, particularly NG, contained higher concentrations of total flavonoids than aged peels. All three peel additives tended to improve growth performance, with 3C showing the most pronounced growth‐promoting effect. Dietary supplementation significantly increased the levels of sweet‐ and umami‐associated amino acids in muscle. The NG group exhibited the highest antioxidant enzyme activities and inflammatory/stress‐related marker expression levels, whereas the 3C group showed the lowest levels of oxidative and inflammatory markers. Intestinal microbiota analysis revealed increased microbial richness and altered community composition in all treatment groups, with 3C notably enhancing microbial diversity and altering taxa abundance patterns. Collectively, these findings suggest that three distinct citrus peels may exert differential functional effects as feed additives, although inclusion level and peel type influence the balance between growth promotion and immune activation. This study provides preliminary evidence supporting the utilization of citrus by‐products in aquafeeds and supports sustainable and circular aquaculture development.

## 1. Introduction

The genus *Citrus* comprises diverse fruit species widely consumed worldwide. *Citrus reticulata* “Gonggan” (GG), a hybrid of *C. reticulata* and *C. sinensis* (L.) Osbeck, is extensively cultivated in southern China [1]. In 2022, production in Deqing County, Guangdong Province, reached 300,000 tons and continues to increase [2]. GG fruits are mainly consumed fresh or processed, whereas the peels are frequently discarded, resulting in resource waste and environmental burdens [3]. *C. reticulata* “’Chachi” (CC) is traditionally used to produce “Chenpi” (CP), a dried peel widely applied in traditional Chinese medicine (TCM). CP exhibits antioxidant, anti‐inflammatory, antimicrobial, and immunomodulatory activities and has documented protective effects on the gut and liver [4–6]. It is currently incorporated into numerous traditional Chinese patent medicines and is also used as a food and feed additive, reflecting its considerable economic value in China [7–10].

The application of TCM as feed additives in China dates back more than 1500 years [11]. Compared with synthetic additives and antibiotics, plant‐derived additives are characterized by low toxicity, biodegradability, and reduced risk of antimicrobial resistance [12]. Increasing evidence indicates that herbal additives can improve growth performance and immune status in livestock and aquatic species [13–15]. Citrus peels are rich in flavonoids, polysaccharides, polyphenols, and terpenoids, which confer antioxidant and immunomodulatory properties [4, 5, 8]. Dietary supplementation with *C. sinensis* peel has been shown to enhance growth performance and immune responses in terrestrial animals [16–18], suggesting its potential application in aquafeeds.

Aquaculture is one of the fastest growing food production sectors globally. Phytogenic feed additives are increasingly explored as alternatives to antibiotics in aquaculture. Herbs such as *Origanum vulgare*, *Cinnamomum cassia* (L.) D. Don, and *Mentha canadensis* L., as well as compound formulations, have been reported to improve flesh quality, disease resistance, and intestinal microbiota composition in species including *Oreochromis niloticus*, *Micropterus salmoides*, and *Oncorhynchus mykiss* [19–22]. For example, cinnamaldehyde derived from *C. cassia* enhances intestinal epithelial integrity, modulates inflammatory responses, and increases the abundance of beneficial bacteria, particularly *Cetobacterium*, in *M. salmoides* [20, 21]. Supplementation with *C. sinensis* peel improves intestinal nutrient absorption in *O. niloticus* and enhances disease resistance in *Silurus asotus* [23–25]. CP has also been reported to improve feed palatability in *Cyprinus carpio*, *Carassius auratus*, and *Channa argus* [26]. While the use of plant‐derived feed additives is well documented in aquaculture, the differential effects of specific citrus by‐product types—particularly discarded GG peel and fresh versus aged CP peel—on growth, flesh quality, and gut microbiota of *O. niloticus* remain underexplored.

*O. niloticus*, an economically important tilapia species, is characterized by rapid growth and high adaptability, making it one of the major cultured species in China. The present study evaluated the effects of discarded GG peel and fresh or aged CC peel as dietary additives on the growth performance, flesh quality, disease resistance, and intestinal microbiota of *O. niloticus*. This work aims to assess the feasibility of utilizing citrus by‐products as functional aquafeed ingredients and to provide experimental evidence to support sustainable aquaculture practices.

## 2. Material and Methods

### 2.1. Plant Material Collection and Authentication

**Figure 1:**
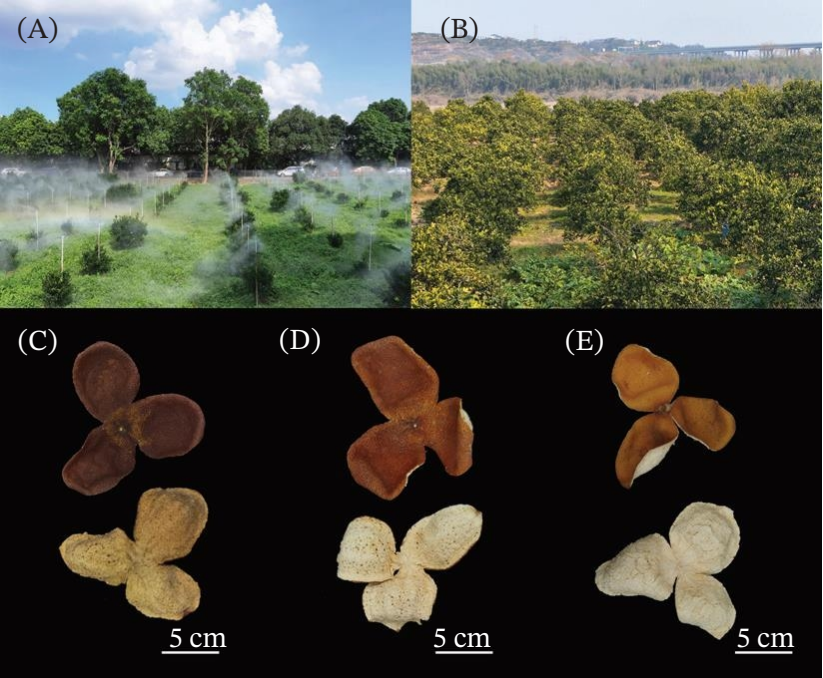
(A) *Citrus reticulata* “Chachi” (CC) orchard in Xinhui District, Jiangmen City, Guangdong Province (113°02’E, 22°31’N); (B) *C. reticulata* “Gonggan” (GG) orchard in Deqing County, Zhaoqing City, Guangdong Province (111°59’E, 23°07’N); (C) 3‐year‐aged CC peel (3C); (D) freshly harvested CC peel (NC); and (E) freshly harvested GG peel (NG). Bar = 5 cm.

The CC fruits were harvested in November 2019 and 2022 from Xinhui District, Jiangmen City, Guangdong Province, China (113°02’E, 22°31’N) (Figure 1A). GG fruits were collected in November 2022 from Deqing County, Zhaoqing City, Guangdong Province, China (111°59’E, 23°07’N) (Figure 1B). The peels were air‐dried at room temperature and stored under dry conditions until they were used. Peels harvested in 2019 and stored until 2022 were designated as 3‐year‐aged CC (3 C) (Figure 1C). Freshly harvested CC and GG peels collected in 2022 were designated as NC (Figure 1D) and NG (Figure 1E), respectively. All plant materials were authenticated by Associate Professor Shejian Liang (South China Agricultural University [SCAU], China).

### 2.2. Major Flavonoids Analysis

Dried peels were pulverized and passed through a 60‐mesh sieve. A 0.35 g sample was extracted with 35 mL of 70% methanol by ultrasonication at 45°C for 40 min (300 W, 40 kHz). The extract was filtered through a 0.22 μm membrane filter and stored at 4°C prior to the analysis.

Flavonoid contents were determined using high‐performance liquid chromatography (HPLC) (Waters, Milford, MA, USA) equipped with a Waters ACQUITY UPLC HSS T3 C18 column (2.1 mm × 100 mm, 1.8 μm). The mobile phase consisted of solvent A (acetonitrile) and solvent B (0.2% formic acid in water). Gradient elution was performed as follows: 0–6 min, 0%–30% A; 6–10 min, 30%–35% A; 10–16 min, 35%–60% A; 16–18 min, 60%–80% A; and 18–22 min, 80%–25% A. The injection volume was 2 μL, the flow rate was 0.70 mL·min^−1^, the column temperature was maintained at 40°C, and detection was conducted at 283 nm [4].

Reference standards of hesperidin (CAS 520‐26‐3), tangeretin (CAS 478‐01‐3), and nobiletin (CAS 481‐53‐8) were obtained from Nanjing Bencaoyikang Biotechnology Co., Ltd. Calibration curves were constructed using six concentration levels based on peak area values, with correlation coefficients (*r* ^2^) greater than 0.99 (Figure S1).

### 2.3. Research on the Growth and Intestinal Microbiota of *O. niloticus*

#### 2.3.1. Experimental Animals

*O. niloticus* (4–6 cm body length and 5.5 ± 0.5 g body weight) were purchased from Guangdong Hengyi Aquaculture Fry Farm, Guangzhou, China. Fish were acclimated and maintained in 600 L indoor recirculating aquaculture tanks. A 12 h light/dark photoperiod was maintained using daylight lamps, and the water temperature was maintained at 30 ± 1°C. Continuous aeration was provided. Feces were removed daily, and 20% of the chlorine‐free water was renewed. Filter materials were replaced every 2 days to maintain the water quality. Fish were fed twice daily (09:00 and 17:00). All procedures were conducted in accordance with the guidelines of the SCAU Animal Experiment Control and Supervision Committee, the UK Animals (Scientific Procedures) Act, 1986, and EU Directive 2010/63. This study was approved by the SCAU Animal Ethics Committee (Number 2022B101).

#### 2.3.2. Preparation of Experimental Feeds

Dried peels (400 g) were cut into 2–3 cm pieces and soaked in 4000 g of distilled water for 30 min. The mixture was boiled in a water bath at 100°C for 30 min. The residue was re‐extracted with 4000 g of distilled water at 100°C for an additional 30 min. The combined filtrates were concentrated to 160 mL by gentle boiling to obtain a decoction (0.25 g mL^−1^, calculated as the dry peel weight). The decoction was aliquoted into amber bottles and stored at 4°C until use.

The basal diet consisted of commercial *O. niloticus* feed (Guangdong Tongwei Aquaculture Feed Co., Ltd., China; 32% crude protein, 6% crude lipid, 12% moisture, 8% ash, and 12% crude fiber; 18.5 MJ·kg^−1^ gross energy). The ingredients included fish meal, soybean meal, wheat flour, corn gluten meal, and a vitamin–mineral premix.

Based on prior empirical findings regarding biomass in studies involving citrus peel‐based additives [23, 26], the dosage level used in this study was determined accordingly. Basal feed (100 g) was evenly sprayed with 100 mL of peel decoction (0.25 g·mL^−1^) using a hand‐held sprayer. This resulted in a final inclusion level of 25 g of peel solids per 100 g feed (dry weight basis). The mixture was thoroughly mixed and air‐dried at 25°C for 3–5 min until fully absorbed. The final moisture content was ~20% and comparable among the groups. To ensure isonitrogenous and isoenergetic experimental conditions, feed allowance was adjusted based on the dry matter content. The control group received an equivalent volume (100 mL) of physiological saline applied to the feed.

#### 2.3.3. Experimental Design

The experiment followed a completely randomized design with a tank as the experimental unit. Healthy fish of uniform size were randomly assigned to four groups. Each treatment consisted of three replicate tanks (*n* = 3), with 10 fish per tank, yielding a total of 12 experimental tanks. The control group received feed treated with physiological saline. The experimental groups included 3C (3‐year‐aged CC peel extract), NC (fresh CC peel extract), and NG (fresh GG peel extract). The feed was provided in submerged feeding trays. Thirty minutes after feeding, uneaten feed was carefully siphoned onto a preweighed 60‐mesh sieve, dried at 60°C to constant weight, and reweighed to calculate daily apparent feed intake per tank (g·tank^−1^·day^−1^). The feed conversion ratio (FCR) was calculated as follows: FCR = total dry feed intake (g)/total weight gain (g) per tank over the experimental period. The feed allowance was adjusted based on dry matter content to ensure isonitrogenous and isoenergetic conditions across treatments. To minimize palatability and intake bias, all diets were prepared from the same batch of basal feed and sprayed with equal volumes (100 mL per 100 g feed) of either a peel decoction or physiological saline. During the first week, feeding behavior was monitored visually; no differential rejection or consumption latency was observed among treatments, indicating comparable feed acceptability. Fish behavior and health status were monitored daily. For growth performance evaluation, the body weight was recorded weekly for all fish (*n* = 30) (Figure S2). On day 71, tissue sampling and biochemical analyses were performed (*n* = 6 per group).

#### 2.3.4. Terminal Sampling and Viscerosomatic Index (VSI)

On day 71, following a 24 h fasting period, six fish per group were randomly selected and euthanized with an overdose of MS‐222. The body weight was recorded prior to dissection. Fish were dissected on ice, and viscera weight was recorded to calculate the VSI. Dorsal muscle (1–2 g), liver, intestine, and mid‐to‐posterior intestinal contents were collected and immediately snap‐frozen in liquid nitrogen for subsequent analyses.

#### 2.3.5. HPLC‐MS/MS Analysis of Free Amino Acids in Muscle

The dorsal muscle (0.5 g) was homogenized in 2 mL of 0.1 mol·L^−1^ HCl. The homogenate was sonicated at 4°C for 40 min with intermittent mixing and centrifuged at 10,000 × *g* for 10 min at 4°C. The supernatant was collected, and the extraction was repeated. Combined supernatants were filtered through a 0.22 μm membrane filter prior to the analysis.

Amino acids were quantified by HPLC‐MS/MS using a Waters ACQUITY UPLC BEH Amide column (1.7 μm, 2.1 mm × 100 mm) maintained at 30°C. The mobile phase consisted of solvent A (20 mmol L^−1^ ammonium formate in water) and solvent B (20 mmol L^−1^ ammonium formate in acetonitrile). The gradient program was as follows: 0–15 min (0%–30% A), 15–16 min (30%–0% A), and 16–30 min (0% A). The injection volume was 5 μL, and the flow rate was 0.25 mL min^−1^. Mass spectrometry was performed using electrospray ionization in the positive mode with multiple reaction monitoring (MRM). The ion source temperature was 450°C, ion spray voltage 5500 V, collision gas set to medium, curtain gas 30 psi, and nebulizer and auxiliary gases at 50 psi. Detailed acquisition parameters are provided in Table S1. Calibration curves were prepared for 20 amino acids (e.g., Ile, Asp, Lys, and Ser) (Figure S3).

#### 2.3.6. Determination of Hepatic Antioxidant Enzyme Activities

The liver tissue (0.1 g) was homogenized in 1 mL extraction buffer on ice and centrifuged (12,000 × *g*, 10 min, 4°C). The supernatant was collected for analysis.

The activities of superoxide dismutase (SOD, WST‐8 method), catalase (CAT), and peroxidase (POD) were determined using commercial assay kits (Guangzhou Hezhong Biotechnology Co., Ltd.) according to the manufacturer’s instructions. Absorbance was measured at 450 nm (SOD), 240 nm (CAT), and 470 nm (POD) using a microplate reader. Enzyme activities were calculated according to the manufacturer’s instructions (Table S2).

#### 2.3.7. qPCR Analysis of Immune‐Related Genes in Liver

Total RNA was extracted from the liver tissue (~0.1 g) using the TRIzol reagent (Thermo Fisher Scientific). RNA was reverse‐transcribed into cDNA according to the manufacturer’s instructions using a commercial kit (Monad Biotechnology Co., Ltd.). qPCR was performed using a commercial kit (QIAGEN) with gene‐specific primers (Table S3). Primer specificity was confirmed by melting curve analysis (a single peak per amplicon). Amplification efficiency (E) ranged from 92% to 108%, with standard curve correlation coefficients *R* ^2^ > 0.98. The stability of the reference gene (β‐actin) was verified across all treatment groups prior to relative quantification using the 2^−ΔΔCt^ method.

#### 2.3.8. 16S rDNA Gene Sequencing of Intestinal Contents

Genomic DNA was extracted from the fish’s intestinal contents. PCR amplification was conducted using the TransGen AP221−02 kit. After detection and purification, sequencing libraries were constructed using a library preparation kit from APExBIO. Libraries were quantified and validated and then sequenced on the Illumina NovaSeq 6000 platform using the PE250 paired‐end strategy to generate raw reads of the 16S rRNA gene amplicons. Raw sequences were quality‐filtered to generate clean reads.

Clean reads were processed using DADA2 to generate amplicon sequence variants (ASVs). Taxonomic annotation was performed in Qiime2 against the Silva‐132‐99 database to determine the microbial community composition. Alpha diversity indices were calculated to assess within‐sample richness and diversity, and beta diversity analysis was conducted to evaluate differences in the microbial community structure among groups. Community composition was visualized using rank‐abundance curves, bar plots, heatmaps, and Krona charts.

### 2.4. Data Analysis

Data were compiled and processed using Excel 2020. Statistical analyses were performed using SPSS 25.0 (IBM Corp., Armonk, NY, USA). Growth performance data (body weight over time) were analyzed using repeated‐measures ANOVA to account for within‐subject correlations across sampling time points. One‐way ANOVA was applied for between‐group comparisons of the endpoint parameters. Statistical significance was set at *p* < 0.05.

## 3. Results and Discussion

### 3.1. Content of Major Flavonoids in Different *Citrus* Peels

**Figure 2:**
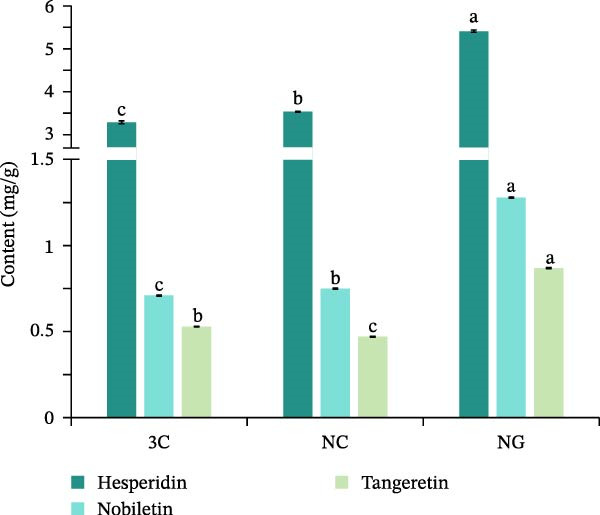
Content of major flavonoid compounds in different *Citrus* peels. 3C represents the 3‐year‐aged *Citrus reticulata* “Chachi” peel, NC represents the fresh *C. reticulata* “Chachi” peel, and NG represents the fresh *C. reticulata* “Gonggan” peel. Lowercase letters “a, b, and c” on the different colored bars indicate significant differences among the samples (*p* < 0.01).

Hesperidin, tangeretin, and nobiletin are major bioactive flavonoids present in citrus peels and have been reported to exhibit immunomodulatory, antibacterial, and antioxidant activities [27]. Among the experimental groups, NG peels contained significantly higher levels of hesperidin (5.41 mg·g^−1^), tangeretin (1.28 mg·g^−1^), and nobiletin (0.87 mg·g^−1^) compared with those in the NC and 3C groups (*p* < 0.01) (Figure 2). These results reveal a correlation between the higher flavonoid content in NG peel and the observed biological responses. However, direct causal evidence linking specific flavonoid compounds to the measured immunomodulatory or antioxidant effects was not established in this study.

NC peels also showed higher hesperidin (3.54 mg·g^−1^) and tangeretin (0.75 mg·g^−1^) contents than 3C (*p* < 0.01), whereas nobiletin was more abundant in 3C (0.53 mg·g^−1^) than in NC (0.47 mg·g^−1^). These findings are consistent with previous reports indicating that hesperidin and tangeretin decrease with increasing aging time [28], while nobiletin gradually accumulates during 1–4 years of aging [29]. Collectively, unaged peels, particularly those from the NG group, retained higher total flavonoid contents than aged peels, suggesting greater potential as functional feed additives with antioxidant and immunomodulatory properties.

### 3.2. Comparison of Body Weight, VSI, and Feed Efficiency of *O. niloticus*

**Figure 3:**
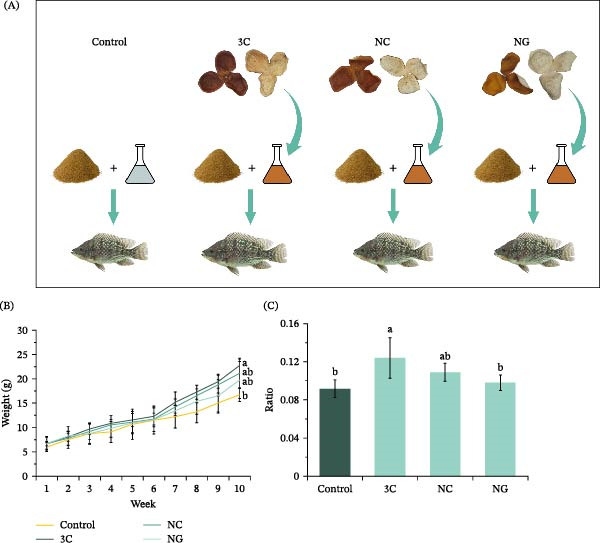
(A) Experimental design; (B) changes in body weight; (C) viscerosomatic index (VSI). Control represents the blank control group, 3C represents the 3‐year‐aged *Citrus reticulata* “Chachi” peel group, NC represents the fresh *C. reticulata* “Chachi” peel group, and NG represents the fresh *C. reticulata* “Gonggan” peel group. Data are shown as mean ± SEM, *n* = 30 in (B) and *n* = 6 in (C). In each subfigure, different lowercase letters “a and b” indicate statistically significant differences (*p* < 0.01).

Experimental design as shown in Figure 3A. The body weight of *O. niloticus* in all four groups increased steadily throughout the experimental period. After week 6, the rate of weight gain in the experimental groups (3 C, NC, and NG) exceeded that of the control group (Figure 3B). By week 10, the mean body weight in the 3C group reached 22.73 g, which was significantly higher than that of the control group (18.68 g) (*p* < 0.01). Although the NC (21.12 g) and NG (19.75 g) groups also showed higher mean body weights than the control group, no significant differences were detected among the three treatment groups (*p* > 0.01). Consistently, the 3C group exhibited the highest specific growth rate (SGR) (2.15 ± 0.08%·day^−1^) and the lowest FCR (1.42 ± 0.05), both significantly improved compared with the control group (SGR: 1.89 ± 0.06%·day^−1^; FCR: 1.68 ± 0.07) (*p* < 0.05) (Table S4). The reduced FCR in the 3C group indicates improved feed utilization efficiency rather than simply increased feed intake.

Improved feed acceptance was observed in fish receiving peel‐supplemented diets, particularly in the NC and 3C groups, which is consistent with previous findings that CP enhances feed palatability in *C. carpio*, *C. auratus*, and *O. argus* [26]. The enhanced growth performance may be associated with improved intestinal nutrient absorption capacity [23, 30], thereby contributing to greater weight gain and metabolic efficiency [25, 31]. However, the surface‐spraying method may affect flavor stability and uniform intake.

The VSI, an indicator of visceral organ development and physiological status in *O. niloticus* [32], was elevated in all treatment groups compared with the control, with statistical significance observed only in the 3C group (*p*  < 0.01) (Figure 3C). The CC groups (3C and NC) showed higher VSI values than the NG group, with a significant difference between 3C and NG (*p*  < 0.01). Notably, the pattern of VSI variation was consistent with that of the body weight and growth performance. Previous studies have shown that increased VSI is associated with enhanced antioxidant capacity, immune function, and nutrient assimilation in fish [33], and similar relationships between elevated VSI and improved growth have been reported in *Labidochromis caeruleus*, *Acipenser schrenckii*, and *Rachycentron canadum* [34, 35]. Collectively, these results suggest that dietary supplementation with citrus peel extracts, particularly 3‐year‐aged CC peel (3 C), promotes growth performance in *O. niloticus*. This effect may be related to enhanced nutrient utilization and visceral development.

### 3.3. Comparison of Amino Acid Composition in Fish Muscle

The contents of crude protein and amino acids in fish muscle are presented in Table 1. No significant differences were observed in crude protein content among the groups. In the control group, the concentrations of Ile, Ser, Met, Val, Pro, Ala, Glu, Leu, Trp, Tyr, Thr, and Phe were significantly higher than those in the three experimental groups (*p* < 0.01). In contrast, the levels of Asp, Lys, Gly, Arg, His, and Asn were significantly lower than those in the experimental groups (*p* < 0.01). Among the three experimental groups, the contents of Asp, Lys, Ser, Gly, and Asn in the 3C group were significantly higher than those in the NC and NG groups (*p* < 0.01). In the NC group, Ile, Met, Val, Pro, Leu, Tyr, Thr, His, and Phe were significantly higher than those in the other two treatment groups (*p* < 0.01). In the NG group, Arg was the only amino acid detected at a significantly higher level compared with the other two groups (*p* < 0.01).

**Table 1:** Content of crude protein and amino acid composition in fish muscle.

	Control	3C	NC	NG
Crude protein (g·kg^−1^)	193.596 ± 0.227	191.789 ± 0.514	193.477 ± 1.249	183.316 ± 9.144
Ile (μg·g^−1^)	76.190 ± 2.786	16.415 ± 0.643 ^∗∗^	33.080 ± 0.325 ^∗∗^	21.265 ± 0.646 ^∗∗^
Asp (μg·g^−1^)	161.215 ± 3.443	500.560 ± 0.891 ^∗∗^	244.045 ± 4.476 ^∗∗^	203.825 ± 2.935 ^∗∗^
Lys (μg·g^−1^)	14.080 ± 0.0424	408.510 ± 1.824 ^∗∗^	24.340 ± 1.075 ^∗∗^	288.905 ± 2.807 ^∗∗^
Ser (μg·g^−1^)	89.370.127	86.365 ± 0.516 ^∗∗^	65.445 ± 0.898 ^∗∗^	63.565 ± 0.573 ^∗∗^
Met (μg·g^−1^)	31.010 ± 0.283	13.810 ± 2.079 ^∗∗^	20.355 ± 0.672 ^∗∗^	15.970 ± 0.707 ^∗∗^
Val (μg·g^−1^)	67.535 ± 0.460	25.375 ± 0.559 ^∗∗^	44.400 ± 0.622 ^∗∗^	31.450 ± 1.641 ^∗∗^
Pro (μg·g^−1^)	127.325 ± 1.096	78.950 ± 2.461 ^∗∗^	109.915 ± 2.636 ^∗∗^	76.670 ± 0.622 ^∗∗^
Ala (μg·g^−1^)	13.620 ± 0.849	10.075 ± 0.615 ^∗∗^	10.110 ± 0.156 ^∗∗^	10.090 ± 0.156 ^∗∗^
Glu (μg·g^−1^)	287.140 ± 6.746	199.920 ± 0.905 ^∗∗^	198.485 ± 0.347 ^∗∗^	104.480 ± 0.042 ^∗∗^
Leu (μg·g^−1^)	22.500 ± 0.735	8.635 ± 0.460 ^∗∗^	15.955 ± 0.205 ^∗∗^	13.025 ± 0.375 ^∗∗^
Gly (μg·g^−1^)	1186.500 ± 9.688	1487.225 ± 21.616 ^∗∗^	1332.001 ± 2.800 ^∗∗^	1361.100 ± 44.124 ^∗∗^
Arg (μg·g^−1^)	18.925 ± 0.120	55.920 ± 3.720 ^∗∗^	92.900 ± 2.235 ^∗∗^	151.430 ± 1.542 ^∗∗^
Cys (μg·g^−1^)	10.195 ± 0.403	9.720 ± 0.424	9.405 ± 0.276	10.080 ± 0.354
Trp (μg·g^−1^)	28.550 ± 0.467	21.090 ± 0.311 ^∗∗^	21.775 ± 0.177 ^∗∗^	22.225 ± 1.393 ^∗∗^
Tyr (μg·g^−1^)	60.685 ± 0.743	25.605 ± 0.262 ^∗∗^	34.880 ± 0.184 ^∗∗^	31.375 ± 1.633 ^∗∗^
Thr (μg·g^−1^)	131.910 ± 0.764	90.625 ± 0.545 ^∗∗^	102.280 ± 0.382 ^∗∗^	64.430 ± 0.636 ^∗∗^
His (μg·g^−1^)	173.635 ± 7.828	403.395 ± 3.203 ^∗∗^	432.985 ± 1.252 ^∗∗^	362.210 ± 1.216 ^∗∗^
Phe (μg·g^−1^)	65.660 ± 0.467	20.095 ± 0.120 ^∗∗^	31.915 ± 1.252 ^∗∗^	27.265 ± 0.912 ^∗∗^
Asn (μg·g^−1^)	14.190 ± 0.396	35.705 ± 0.601 ^∗∗^	18.280 ± 0.495 ^∗∗^	27.625 ± 1.068 ^∗∗^

*Note:* Control represents the blank control group, 3C represents the 3‐year‐aged *Citrus reticulata* “Chachi” peel group, NC represents the fresh *C. reticulata* “Chachi” peel group, and NG represents the fresh *C. reticulata* “Gonggan” peel group. The symbol “ ^∗∗^" indicate statistically significant differences.

^∗^(*p* < 0.05).

^∗∗^(*p* < 0.01).

Previous studies have demonstrated that individual amino acids contribute to specific flavor attributes in muscle tissue. For example, Gly is associated with sweetness, whereas Asp contributes to umami taste [36]. According to the present results (Table 1), the levels of Gly and Asp in the muscle of fish from the experimental groups (3C, NC, and NG) were significantly higher than those in the control group. Numerous studies have reported that herbal feed additives can improve the muscle texture and sensory quality in fish [37]. Therefore, dietary supplementation with 3C, NC, and NG peels may enhance the sweetness and umami‐related flavor characteristics of fish muscle.

### 3.4. Comparison of Antioxidant Enzyme Activity in Liver

**Figure 4:**
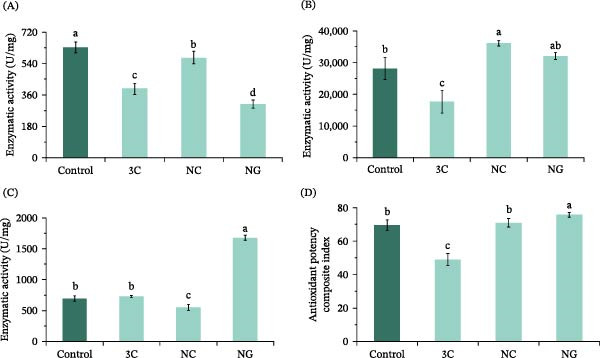
Antioxidant enzyme activity in liver extracts. (A) SOD activity, (B) CAT activity, (C) POD activity, (D) antioxidant potency composite index (APCI). Control represents the blank control group, 3C represents the 3‐year‐aged *Citrus reticulata* “Chachi” peel group, NC represents the fresh *C. reticulata* “Chachi” peel group, and NG represents the fresh *C. reticulata* “Gonggan” peel group. In each subfigure, different lowercase letters “a, b, c, and d” indicate statistically significant differences (*p* < 0.01).

The antioxidant capacity of an organism is closely associated with stress resistance, reactive oxygen species (ROS) scavenging, and maintenance of cellular homeostasis. SOD, CAT, and POD are key antioxidant enzymes in fish [38]. The hepatic antioxidant enzyme activities of *O. niloticus* are presented in Figure 4A–D.

Compared with the control group, SOD activity in the 3C, NC, and NG groups was significantly reduced (*p* < 0.01), with activity levels ranking as NC > 3C > NG (Figure 4A). For CAT activity, the NC and NG groups showed higher values, with the NC group being significantly higher than the Control group, whereas the 3C group was significantly lower than the Control group (*p* < 0.01) (Figure 4B). In terms of POD activity, the NG group was significantly higher than the control group, while the 3C group was significantly lower (*p* < 0.01) (Figure 4C).

SOD, CAT, and POD function synergistically to neutralize ROS and maintain the intracellular redox balance [39]. Previous studies have shown that antioxidant enzyme activities and related gene expression are often upregulated in fish under stress conditions [40]. In the present study, the antioxidant potency composite index (APCI) was applied to evaluate overall antioxidant status [41]. The APCI was significantly higher in the NG group than in the other groups, whereas the NC group showed a slight but nonsignificant increase compared with the control group, and the 3C group exhibited a significantly lower APCI than the control group (*p* < 0.01) (Figure 4D).

Notably, the NG group exhibited relatively high CAT and POD activities and the highest APCI but showed comparatively lower growth performance, suggesting that the elevated enzymatic activities may reflect a compensatory response to oxidative or inflammatory stress rather than enhanced antioxidant efficiency. This interpretation is supported by the upregulated expression of stress‐related (e.g., HSP70) and proinflammatory genes observed in the NG group. However, direct markers of oxidative damage (e.g., lipid or protein oxidation products) were not measured. Therefore, the presence and extent of oxidative injury cannot be conclusively determined, which represents a limitation of the present study. Further investigations incorporating controlled feed intake assessment and histopathological or oxidative damage analyses are warranted to clarify the relationship between antioxidant status and growth performance.

### 3.5. Expression Patterns of Inflammatory and Stress‐Related Genes in Liver

**Figure 5:**
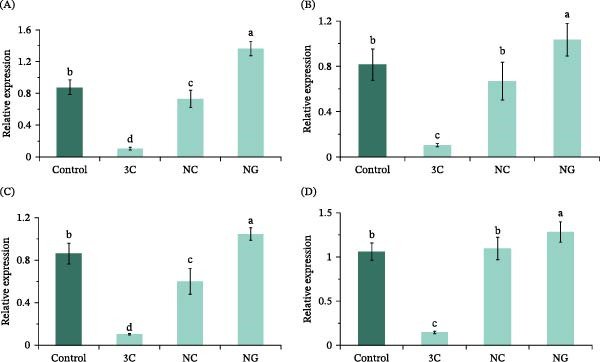
Relative expression of immune‐related genes in liver extracts. (A) IL‐1β gene expression level; (B) TNF‐α gene expression level; (C) IFN‐γ gene expression level; (D) HSP70 gene expression level. Control represents the blank control group, 3C represents the 3‐year‐aged *Citrus reticulata* “Chachi” peel group, NC represents the fresh *C. reticulata* “Chachi” peel group, and NG represents the fresh *C. reticulata* “Gonggan” peel group. In each figure, different lowercase letters “a, b, c, and d” indicate statistically significant differences (*p* < 0.01).

The relative expression levels of immune‐related genes in the liver of *O. niloticus* are presented in Figure 5A–D. Compared with the control group, hepatic IL‐1β, TNF‐α, IFN‐γ, and HSP70 expression levels were significantly downregulated in the 3C group (*p* < 0.01), representing the lowest values among all treatments. In the NC group, IL‐1β and IFN‐γ expression levels were significantly reduced compared with the control group (*p* < 0.01), whereas TNF‐α and HSP70 expression showed no significant differences. In contrast, all four genes (IL‐1β, TNF‐α, IFN‐γ, and HSP70) were significantly upregulated in the NG group relative to the control group (*p* < 0.01), exhibiting the highest expression levels among the four groups. Overall, the expression pattern of immune‐related genes followed the order NG > NC > 3 C.

Previous studies have reported that CP extracellular vesicles can suppress inflammatory mediators, including NO, IL‐6, IL‐1β, and TNF‐α [5, 25]. TCM herbs have also been shown to modulate immune function through both specific and nonspecific immune pathways [13, 14, 17, 25, 42, 43]. The markedly elevated expression of proinflammatory cytokines (IL‐1β, TNF‐α, and IFN‐γ) and the stress marker HSP70 in the NG group indicates activation of inflammatory and cellular stress responses, rather than necessarily representing beneficial immune enhancement. Sustained upregulation of these markers may reflect suboptimal physiological conditions or mild inflammatory stress [44]. This interpretation is consistent with the comparatively lower weight gain observed in the NG group relative to the 3C treatment.

While NG peel supplementation was associated with enhanced immune gene expression and antioxidant enzyme activity, a direct causal relationship between these molecular responses and growth performance cannot be established in the absence of complementary evidence (e.g., histopathological assessment, pathogen challenge tests, or quantitative feed intake analysis). Therefore, NG peel appears to possess immunomodulatory potential. However, optimization of dietary inclusion levels is necessary to balance immune stimulation with growth efficiency in practical aquaculture applications.

### 3.6. Analysis and Comparison of 16S rDNA Gene Sequencing of Intestinal Microbiota

#### 3.6.1. Diversity of Intestinal Microbiota

**Figure 6:**
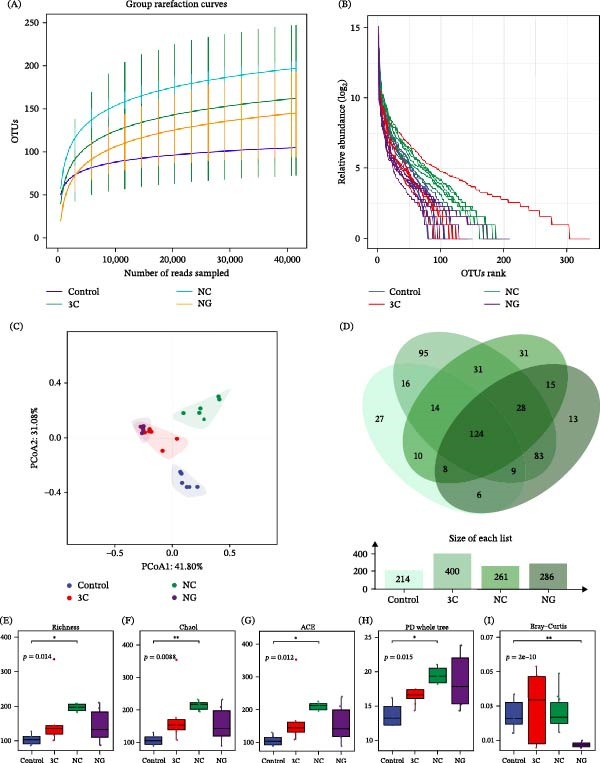
(A) Sample dilution curve. (B) Rank abundance plot of the sample. (C) PCoA analysis plot of the sample. (D) Venn diagram. (E–H) Boxplots of alpha‐diversity indices of the sample, including richness, Chao1, ACE, and PD whole tree. (I) Boxplot of beta‐diversity analysis based on UniFrac distance. Control represents the blank control group, 3C represents the 3‐year‐aged *Citrus reticulata* “Chachi” peel group, NC represents the fresh *C. reticulata* “Chachi” peel group, and NG represents the fresh *C. reticulata* “Gonggan” peel group. The symbols “ ^∗^” and “ ^∗∗^” indicate statistically significant differences,  ^∗^(*p* < 0.05),  ^∗∗^(*p* < 0.01).

Microbial diversity analysis provides insights into the abundance and composition of intestinal microbial communities, with α‐ and β‐diversity indices commonly used to quantify species richness and community structure [13]. As shown in Figure 6A, the rarefaction curves for all samples reached a plateau, indicating that the sequencing depth was sufficient to capture the majority of microbial diversity [13, 45]. The rank abundance curves (Figure 6B) reveal relatively small differences in ASV abundance within groups, suggesting uniform community composition. Principal coordinates analysis (PCoA) (Figure 6C) demonstrates modest differences among the four groups, indicating broadly similar gut microbiota structures. The Venn diagram (Figure 6D) shows that the three experimental groups harbored more ASVs than the control group, with the 3C group exhibiting the highest total (400) and unique ASVs (95), suggesting enhanced microbial richness in this treatment.

Analysis of α‐diversity indices (Figure 6E–H) revealed that richness, Chao1, ACE, and PD whole tree values were significantly higher in the experimental groups compared with the control group (*p* < 0.05), indicating increased species richness, more comprehensive representation of rare taxa, and greater phylogenetic diversity [46, 47]. In β‐diversity analysis based on Bray–Curtis distances (Figure 6I), the NG group showed significantly lower dispersion than the Control group (*p* < 0.01), reflecting reduced inter‐individual variation and a more homogeneous gut microbiota structure, whereas the 3C group exhibited significantly higher dispersion (*p* < 0.01), indicating greater inter‐individual variability and more heterogeneous microbial communities. It is noteworthy that PCoA plots (Figure 6C) show overlapping clusters, suggesting that although statistically significant differences exist in community composition, effect sizes are modest, and group centroids are not strongly separated. This suggests that dietary treatments induced subtle shifts rather than dramatic restructuring of the gut microbiota.

Overall, the results indicate that the experimental diets, particularly the 3C treatment, enhanced gut microbial richness and diversity relative to the control group. A diverse and rich gut microbiota is generally considered beneficial as it contributes to microbial community stability, suppresses opportunistic pathogens, and supports nutrient absorption. Furthermore, a highly diverse microbiota can provide a wide range of metabolic functions and participate in physiological processes such as digestion, nutrient assimilation, and immune regulation [19–22, 48].

#### 3.6.2. Taxonomic Analysis of Intestinal Microbiota

The gut is the primary site for digestion and nutrient absorption, and its microbial composition is critical for maintaining intestinal stability and host health [13, 49]. We analyzed the relative abundance of microbial taxa in the four groups from the phylum to genus levels.

At the phylum level (Figure 7A), Actinobacteriota, Proteobacteria, Bacteroidota, and Firmicutes were the dominant bacterial phyla across all groups, collectively accounting for over 75% of the total sequences. Actinobacteriota alone exceeded 50% in all groups, reaching more than 75% in the NG and 3C groups, consistent with previous studies [13, 50]. As shown in Figure 7B, the relative abundance of Actinobacteriota was significantly higher in the 3C and NG groups compared with the control group (*p* < 0.01), with the NG group exhibiting the largest increase. Conversely, Proteobacteria and Bacteroidota abundances were significantly lower in all experimental groups relative to the control group (*p* < 0.01). Firmicutes abundance increased significantly in the 3C and NC groups compared with the control group (*p* < 0.01). Previous studies have reported that Firmicutes abundance is higher in cultured crucian carp than in wild populations (*p* < 0.05), and the Firmicutes/Bacteroidota (F/B) ratio is positively associated with energy harvest efficiency [13, 51, 52]. In the present study, all experimental groups exhibited elevated F/B ratios, which is consistent with the observed weight gain in tilapia.

Figure 7(A) Bar chart of species composition at the phylum level. (B) Kruskal–Wallis analysis at the phylum level. (C) Bar charts of species composition at the family and genus levels. (D) Distribution of LDA values for differentially abundant species obtained from LEfSe analysis. Control represents the blank control group, 3C represents the 3‐year‐aged *Citrus reticulata* “Chachi” peel group, NC represents the fresh *C. reticulata* “Chachi” peel group, and NG represents the fresh *C. reticulata* “Gonggan” peel group. The symbol “ ^∗∗^” indicate statistically significant differences, (*p* < 0.01).

**Figure:**
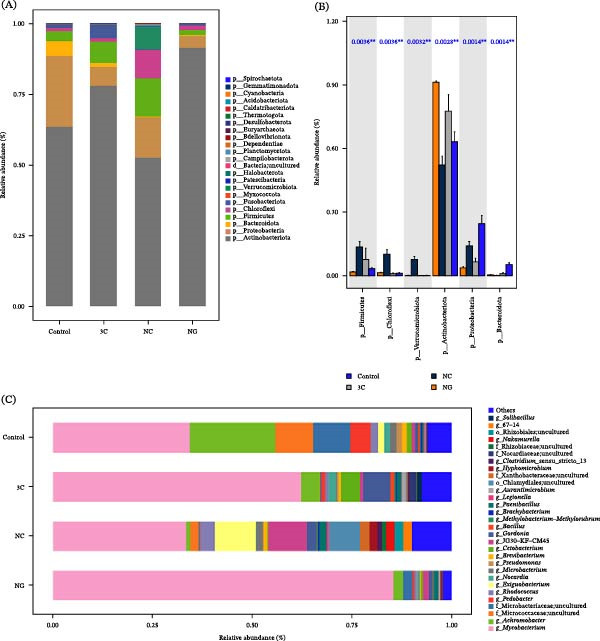

**Figure:**
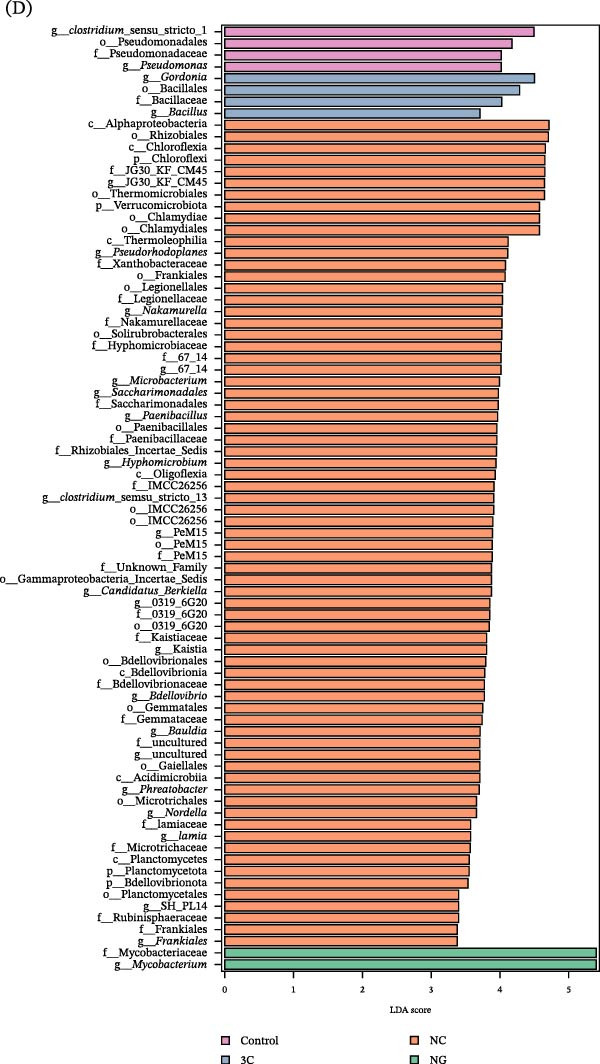

At the family and genus levels (Figure 7C), *Mycobacterium*, *Achromobacter*, *Micrococcaceae*, and *Microbacteriaceae* were the predominant taxa in the gut of *O. niloticus*. The abundance of *Mycobacterium* was significantly higher in the 3C and NG groups compared with the control group (*p* < 0.01), with the NG group showing the largest increase. Mycobacterium is functionally heterogeneous, encompassing both potential fish pathogens (e.g., *M. marinum*) and environmental saprophytes involved in nutrient cycling [50, 53, 54]. Therefore, increased *Mycobacterium* abundance cannot be interpreted as definitively beneficial or pathogenic without further functional characterization. The abundances of *Achromobacter*, *Micrococcaceae*, and *Microbacteriaceae* were significantly reduced in all experimental groups (*p* < 0.01). Previous studies indicate that *Achromobacter*, including *A. pulmonis*, can have pathogenic potential [55, 56], *Micrococcaceae* is often dominant in healthy fish [57] but may be associated with dysbiosis under certain conditions [58], and *Microbacteriaceae* functions are environment‐ and strain‐dependent [59].

Overall, the experimental diets induced shifts in the microbial community structure, with increased abundance of certain taxa (e.g., Firmicutes and Actinobacteriota) and decreased abundance of others (e.g., Proteobacteria and Bacteroidota). 16S rRNA gene sequencing revealed taxonomic shifts. However, functional predictions based on taxonomic annotations remain speculative without metagenomic or metabolomic validation. Therefore, 16S rRNA gene‐based taxonomic profiling alone cannot reliably predict functional outcomes or pathogenic potential, particularly for taxonomically diverse genera such as *Mycobacterium*.

#### 3.6.3. Linear Discriminant Analysis Effect Size (LEfSe) Analysis

LEfSe analysis was conducted to identify microbial taxa associated with treatment‐induced differences in gut community composition following supplementation with NG peel, NC peel, and 3C peel. The cladogram revealed 81 potential microbial biomarkers with significant differences among groups (Figure 7D). In the NC group, 71 genera were significantly enriched relative to the control group, including Alphaproteobacteria, Rhizobiales, and Oligoflexia.

Alphaproteobacteria may influence host metabolic health through metabolites such as short‐chain fatty acids, bile acids, and endocannabinoids. These metabolites act on host receptors and signaling pathways, affecting intestinal barrier function, gut hormone secretion, and overall intestinal physiology [60]. Some members also participate in gut nutrient metabolism, including amino acid turnover, potentially impacting host amino acid levels and glucose tolerance [61, 62]. Oligoflexia contributes to the breakdown of dietary fiber and the production of short‐chain fatty acids such as butyrate, propionate, and acetate, which serve as energy sources for intestinal epithelial cells, support gut barrier integrity, and modulate host immune responses [63]. Bdellovibrionales may prey on harmful bacteria, helping maintain the microbial balance and intestinal health [63]. Bauldia has been reported to alleviate inflammation and metabolic disorders [64]. *Mycobacterium* is involved in microbial interactions that influence gut stability and may affect tight junction protein expression and gut immune cell activity, thereby contributing to barrier function regulation [65].

In the NG group, the abundances of *Mycobacteriaceae* and *Mycobacterium* were significantly increased relative to the control group (*p* < 0.01). In the 3C group, *Gordonia*, Bacillales, Bacillaceae, and *Bacillus* were significantly enriched (*p* < 0.01). *Gordonia* can degrade biomacromolecules and exhibit functions such as antioxidant activity, immune modulation, and cancer prevention [66]. Bacillales, Bacillaceae, and *Bacillus* species contribute to metabolic regulation, reduce host exposure to toxins, and modulate immunity [67, 68]; certain *Bacillus* species (e.g., *B. coagulans* and *B. subtilis*) act as probiotics to promote gut microbial balance [69].

Overall, LEfSe analysis indicates that the experimental diets, particularly NC and 3C treatments, enriched specific microbial taxa that are potentially associated with beneficial functions, suggesting that these peel extracts can positively modulate the gut microbiota of *O. niloticus*.

## 4. Conclusion

This study systematically evaluated the effects of three types of Citrus peels as aquaculture feed additives on the growth performance, flesh quality, antioxidant enzyme activities, inflammatory marker expression, and intestinal microbiota of *O. niloticus*. Key findings are summarized as follows: The contents of major flavonoids, including hesperidin, tangeretin, and nobiletin, were the highest in unaged peels, particularly in NG, compared with aged peels, providing a biochemical basis for differential bioactivity. Dietary supplementation with 3C peel extract significantly promoted weight gain and visceral development in *O. niloticus*. All peel‐supplemented diets enhanced the levels of sweet and umami amino acids in the fish muscle, indicating improved flesh flavor. The NG group exhibited the highest APCI and elevated expression of inflammatory/stress‐related markers (IL‐1β, TNF‐α, IFN‐γ, and HSP70), whereas the 3C group showed the lowest values. These elevated markers in the NG group indicate inflammatory activation or cellular stress rather than improved health or immune function, and whether NG peel possesses immunomodulatory activity requires further validation through challenge studies. All peel extracts increased gut microbiota abundance, with the 3C treatment significantly enhancing the species richness and diversity of specific gut microbial taxa, indicating its potential for establishing and stabilizing intestinal microbial communities. Overall, this study provides preliminary, descriptive evidence suggesting that discarded GG peels and TCM‐derived CP can serve as functional feed additives, with 3C showing superior effects on growth, gut microbiota, and flesh quality. These findings provide a theoretical and practical basis for optimizing *O. niloticus* feed formulations, improving aquaculture efficiency, and promoting high‐value utilization of agricultural by‐products. The results also support sustainable aquaculture practices and enhanced circular utilization of agricultural resources, contributing to eco‐friendly production and the harmonious coexistence of aquaculture and the environment.

## Author Contributions

**Yang Yang:** conceptualization, conceived and designed the experiment, performed the experiment, analyzed the data, writing – original draft preparation. **Zhanqian Wang**: conceived and designed the experiment, performed the experiment, analyzed the data, writing – original draft preparation. **Yifan Hao, Chong Yang, Sisi Xie, Xingchen Li, Nuoying Li, Xiaoxuan Jiang, and Wenbin Zhang**: performed the experiment. **Shejian Liang**: conceptualization, conceived and designed the experiment, writing – review and editing, funding acquisition.

## Funding

This work was supported by the National Natural Science Foundation of China Project (Grant 31570180), the Guangdong Provincial Natural Science Foundation Project (Grant 2025A1515012280), the Guangdong Province Agricultural Standardization Project (Grant 20160907), and the Entrusted Projects by Enterprises (Grant h20210923).

## Ethics Statement

All animal protocols were approved by the Animal Administration and Ethics Committee of the Academic Committee of South China Agricultural University (2022B101) and did not involve any human participants.

## Conflicts of Interest

The authors declare no conflicts of interest.

## Supporting Information

Additional supporting information can be found online in the Supporting Information section.

## Supporting information


**Supporting Information** Figure S1: The standard curve of flavone. Figure S2: Ten‐week records of feeding *Oreochromis niloticus* with different feed additives. Control represents the blank control group, 3C represents the three‐year aged *Citrus reticulata* “Chachi” peel group, NC represents the fresh *C. reticulata* “Chachi” peel group, and NG represents the fresh *C. reticulata* “Gonggan” peel group. Figure S3: The standard curve for 20 types of amino acids. Table S1: Sampling parameter table. Table S2: Detection of antioxidant enzyme activity in the liver. Table S3: Primer information. Table S4: Growth performance parameters.

## Data Availability

The data are available in the article’s Supporting Information.
