# Supplementary material for: Effects of Three Dried Citrus Peels as Feed Additives on Growth Performance and Intestinal Microbiota of Oreochromis niloticus
Source: Aquac Nutr. 2026 Jul 9;2026:5579376. doi: 10.1155/anu/5579376 (PMC13351333; doi:10.1155/anu/5579376)
Supplement: Supplementary file 1 — Supporting Information Figure S1: The standard curve of flavone. Figure S2: Ten‐week records of feeding Oreochromis niloticus with different feed additives. Control represents the blank control group, 3C represents the three‐year aged Citrus reticulata “Chachi” peel group, NC represents the fresh C. reticulata “Chachi” peel group, and NG represents the fresh C. reticulata “Gonggan” peel group. Figure S3: The standard curve for 20 types of amino acids. Table S1: Sampling parameter table. Table S2: Detection of antioxidant enzyme activity in the liver. Table S3: Primer information. Table S4: Growth performance parameters. [file ANU-2026-5579376-s001.doc]

**Effects of three dried *Citrus* peels as feed additives on growth performance and intestinal microbiota of *Oreochromis niloticus***

**Supplementary Materials**


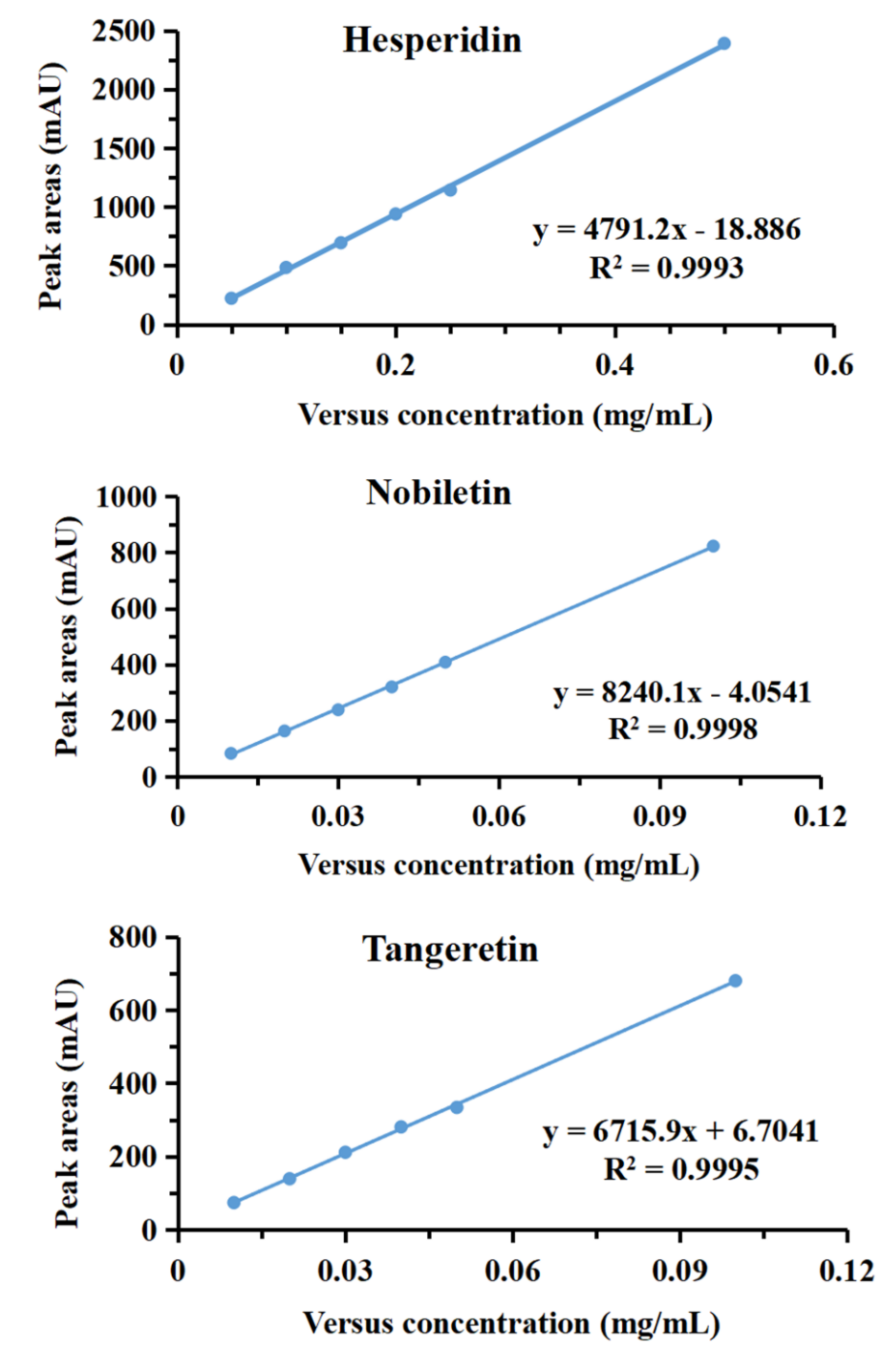


**Figure S1.** The standard curve of flavone.


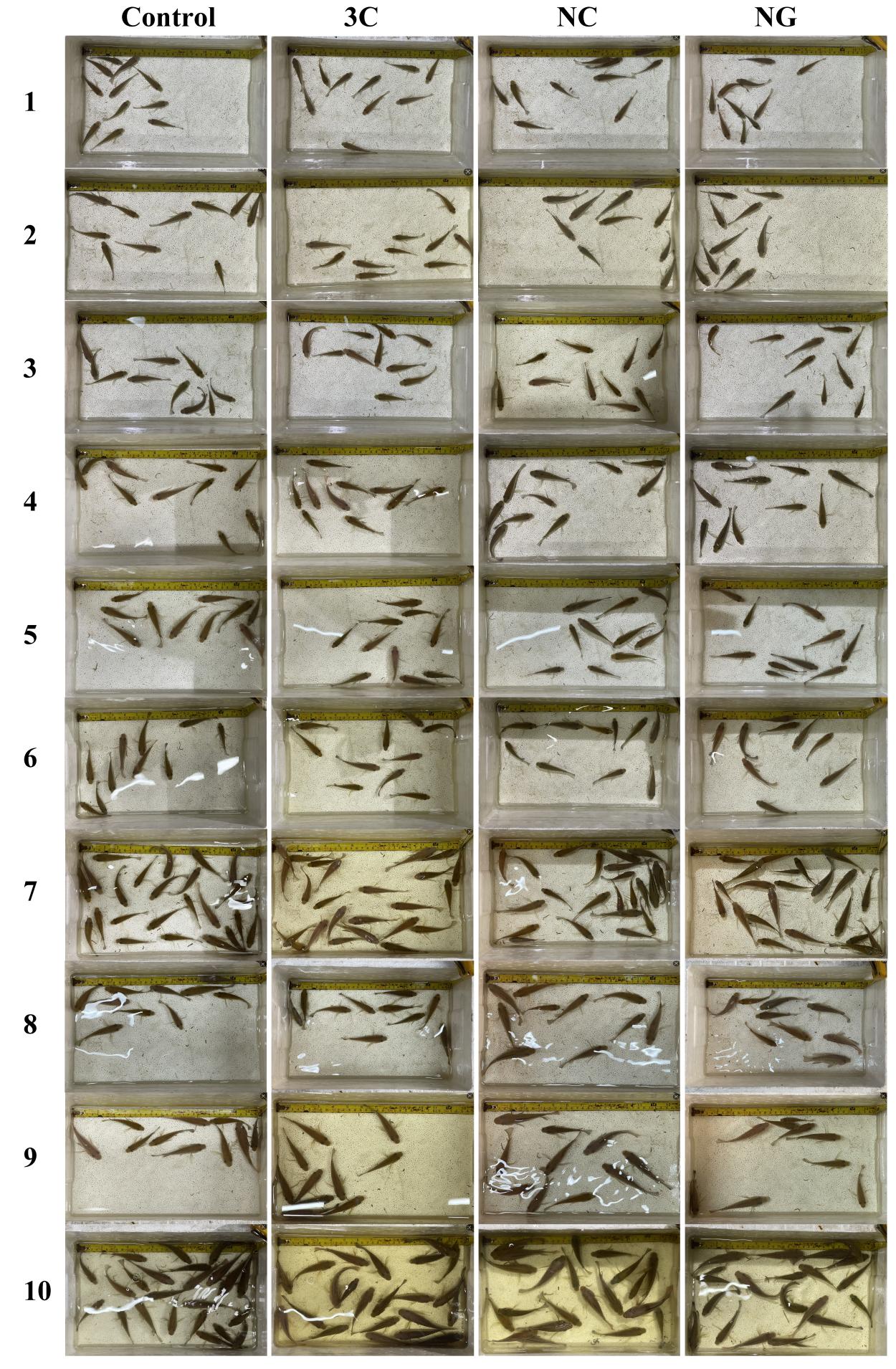


**Figure S2** Ten-week records of feeding *Oreochromis niloticus* with different feed additives. Control represents the blank control group, 3C represents the three-year aged *Citrus reticulata* 'Chachiensis' peel group, NC represents the fresh *C. reticulata* 'Chachiensis' peel group, and NG represents the fresh *C. reticulata* 'Gong Gang' peel group.


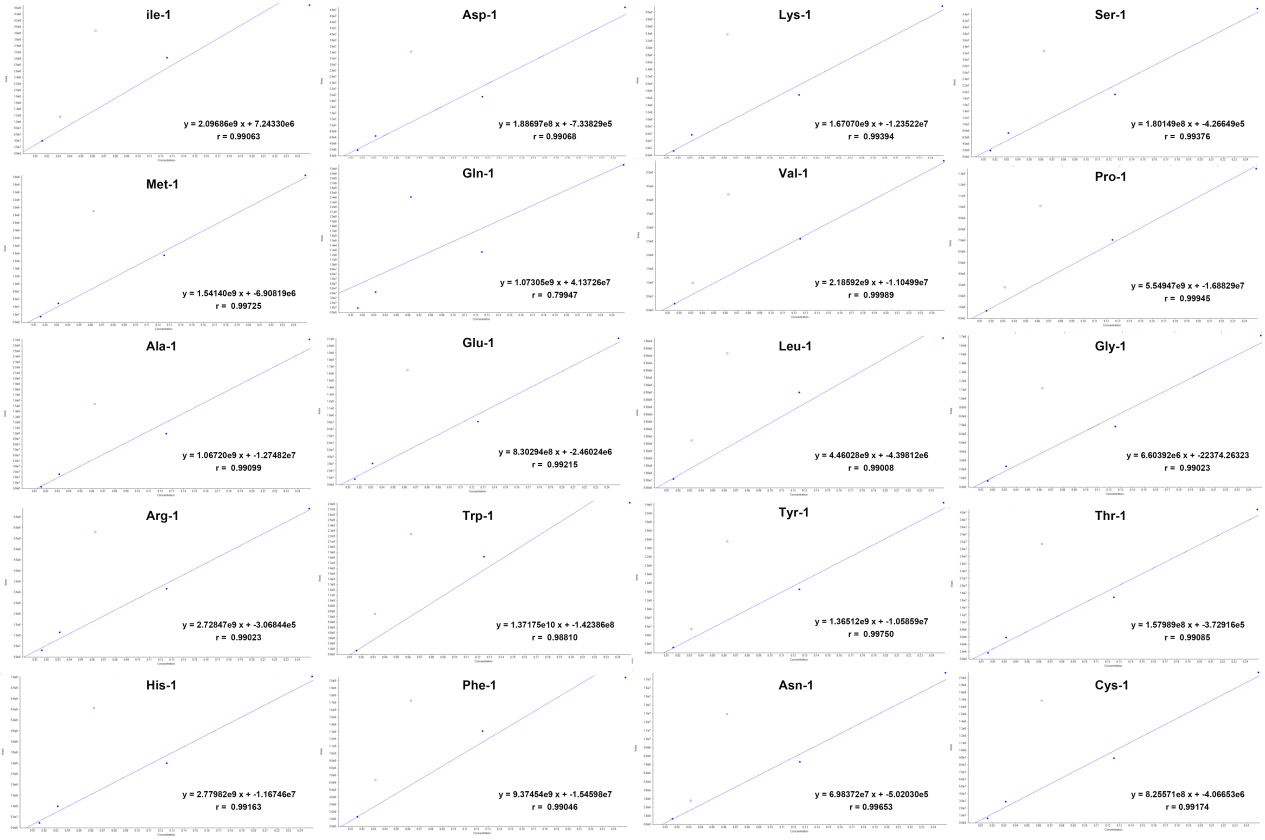


**Figure S3.** The standard curve for 20 types of amino acids.

**Table S1.** Sampling parameter table.

| number | Parent Ion | Daughter Ion | Dwell Time（ms） | Name | DP | CE |
| --- | --- | --- | --- | --- | --- | --- |
| 1 | 132.1 | 86.4 | 50 | Ile-1 | 40 | 14 |
| 2 | 132.1 | 103.8 | 50 | Ile-2 | 40 | 11 |
| 3 | 133.9 | 74.1 | 50 | Asp-1 | 30 | 19 |
| 4 | 133.9 | 88.0 | 50 | Asp-2 | 40 | 13 |
| 5 | 147.1 | 84.3 | 50 | Lys-1 | 30 | 22 |
| 6 | 147.1 | 130.2 | 50 | Lys-2 | 17 | 14 |
| 7 | 106.2 | 60.2 | 50 | Ser-1 | 23 | 16 |
| 8 | 106.2 | 88.2 | 50 | Ser-2 | 6 | 13 |
| 9 | 150.1 | 132.9 | 50 | Met-1 | 33 | 13 |
| 10 | 150.1 | 104.3 | 50 | Met-2 | 34 | 15 |
| 11 | 147.1 | 130.1 | 50 | Gln-1 | 29 | 14 |
| 12 | 147.1 | 84.1 | 50 | Gln-2 | 24 | 23 |
| 13 | 118.1 | 72.1 | 50 | Val-1 | 23 | 15 |
| 14 | 118.1 | 89.7 | 50 | Val-2 | 23 | 9 |
| 15 | 116.2 | 70.1 | 50 | Pro-1 | 34 | 21 |
| 16 | 116.2 | 59.9 | 50 | Pro-2 | 34 | 15 |
| 17 | 90.8 | 65.0 | 50 | Ala-1 | 234 | 26 |
| 18 | 90.8 | 72.9 | 50 | Ala-2 | 94 | 12 |
| 19 | 148.2 | 84.2 | 50 | Glu-1 | 15 | 20 |
| 20 | 148.2 | 102.1 | 50 | Glu-2 | 20 | 14 |
| 21 | 132.1 | 85.9 | 50 | Leu-1 | 19 | 14 |
| 22 | 132.1 | 104.1 | 50 | Leu-2 | 53 | 10 |
| 23 | 76.0 | 30.0 | 50 | Gly-1 | 40 | 14 |
| 24 | 76.0 | 46.0 | 50 | Gly-2 | 40 | 17 |
| 25 | 175.2 | 70.1 | 50 | Arg-1 | 32 | 26 |
| 26 | 175.2 | 116.2 | 50 | Arg-2 | 50 | 19 |
| 27 | 205.1 | 188.3 | 50 | Trp-1 | 16 | 15 |
| 28 | 205.1 | 86.7 | 50 | Trp-2 | 20 | 14 |
| 29 | 182.1 | 165.1 | 50 | Tyr-1 | 35 | 14 |
| 30 | 182.1 | 136.2 | 50 | Tyr-2 | 20 | 19 |
| 31 | 120.1 | 102.1 | 50 | Thr-1 | 35 | 12 |
| 32 | 120.1 | 74.0 | 50 | Thr-2 | 32 | 15 |
| 33 | 156.0 | 110.1 | 50 | His-1 | 41 | 20 |
| 34 | 156.0 | 95.2 | 50 | His-2 | 25 | 21 |
| 35 | 166.1 | 120.1 | 50 | Phe-1 | 44 | 18 |
| 36 | 166.1 | 148.9 | 50 | Phe-2 | 41 | 13 |
| 37 | 133.1 | 87.2 | 50 | Asn-1 | 38 | 14 |
| 38 | 133.1 | 116.0 | 50 | Asn-2 | 49 | 14 |
| 39 | 241.1 | 152.0 | 50 | Cys-1 | 33 | 17 |
| 40 | 241.1 | 120.1 | 50 | Cys-2 | 33 | 24 |

Table S2. Detection of antioxidant enzyme activity in the liver.

| Enzyme Activity | Formula |
| --- | --- |
| SOD | SOD (U/g) = [Inhibition Percentage ÷ ( 1 - Inhibition Percentage) × V_2_] ÷ ( W × V_1_ ÷ V ) × D  Notes: V: Volume of the extraction solution added; V_1_: Volume of the sample added to the reaction system; V_2_: Total volume of the reaction system; D: Dilution factor of the sample; W: Mass of the sample. |
| CAT | CAT (U/g) = [( A_1_ - A_2_ ) × V_1_ ÷ ( ε × d ) × 10^6^ ] ÷ ( W × V_2_ ÷ V_3_ ) ÷ T  Notes: A_1_: Initial absorbance; A_2_: Absorbance after 1 minute; V_1_: Total volume of the reaction system; V_2_: Volume of the sample; V_3_: Volume of the extraction solution; ε: Molar extinction coefficient of H₂O₂, 43.6 L/mol/cm; d: Optical path length of the 96-well plate, 1 cm; T: Reaction time; W: Mass of the sample. |
| POD | POD (U/g) = (A2 - A1) × V ÷ ( W × V2 ÷ V1 ) ÷ 0.005 ÷ T  Notes: A1: Initial absorbance; A2: Absorbance after 1 minute; V: Total volume of the reaction system; V1: Volume of the extraction solution; V2: Volume of the sample; T: Reaction time; W: Mass of the sample. |

All the samples tested above were fresh samples.

**Table S3 Primer information.**

| Primer Name | Sequence (5’ → 3’) |
| --- | --- |
| TNF-α - F | 5’-TCTGGAGTGGAGGAATGGTCAAGG-3’ |
| TNF-α - R | 5’-TGAAGGACGCCTGGCTGTAGAC-3’ |
| IL-1β - F | 5’-ACAAGGATGACGACAAGCCAACC-3’ |
| IL-1β - R | 5’-GGACAGACATGAGAGTGCTGATGC-3’ |
| IFN-γ - F | 5’-GAAACAACTGCCCACTCCGAGTC-3’ |
| IFN-γ - R | 5’-TGCCTGGTAGCGAGCCTGAG-3’ |
| Hsp70 - F | 5'-GCTCTGAACCCCAGCAACACT-3' |
| Hsp70 - R | 5'-TTGTCCTCCCCTTTGTACTCCA-3' |
| β-actin - F | 5’- AAGGACCTGTACGCCAACAC-3’ |
| β-actin - R | 5’- ACATCTGCTGGAAGGTGGAC-3’ |

**Table S4 Growth performance parameters**

| Parameter | Control | 3C | NC | NG |
| --- | --- | --- | --- | --- |
| Initial weight (g) | 5.52 ± 0.48 | 5.48 ± 0.51 | 5.55 ± 0.45 | 5.50 ± 0.50 |
| Final weight (g) | 18.68 ± 1.20b | 22.73 ± 1.45a | 21.12 ± 1.35ab | 19.75 ± 1.28ab |
| Weight gain (g) | 13.16 ± 1.15b | 17.25 ± 1.38a | 15.57 ± 1.25ab | 14.25 ± 1.20ab |
| SGR (%/day) | 1.89 ± 0.06b | 2.15 ± 0.08a | 2.05 ± 0.07ab | 1.95 ± 0.06a |
| FCR | 1.68 ± 0.07a | 1.42 ± 0.05b | 1.52 ± 0.06ab | 1.62 ± 0.07 |
| Survival rate (%) | 96.7 ± 3.3 | 98.3 ± 1.7 | 96.7 ± 3.3 | 95.0 ± 5.0 |

Note: Values are mean ± SEM (n = 30). Different superscripts in the same row indicate significant differences (p < 0.05). Feed Conversion Ratio (FCR) and Specific Growth Rate (SGR) were calculated as follows:

FCR = Total feed intake (g, dry weight) / Weight gain (g)

SGR (%/day) = [(ln Wt - ln W0) / t] × 100

W_t_ is final body weight, W_0_ is initial body weight, and t is feeding duration.
